# Cognitive Emotion Regulation in Early Adolescents with ADHD: Sex Differences in Adaptive Strategies

**DOI:** 10.21203/rs.3.rs-10882093/v1

**Published:** 2026-10-15

**Authors:** Annah R. Cash, Olivia Baron, Melissa C. Miller, Rosanna Breaux, Stephen P. Becker

## Abstract

Emotion regulation (ER) difficulties are common among youth with attention-deficit/hyperactivity disorder (ADHD), yet little is known about the cognitive strategies they use to regulate emotions or whether these associations differ by sex. We examined adaptive and maladaptive cognitive ER in 341 early adolescents (ages 10–12; *M* = 10.90; *SD* = 0.80), 47.8% of whom met diagnostic criteria for ADHD and 52.2% of whom were female. ADHD diagnosis was established using a semi-structured diagnostic interview administered to caregivers, and adolescents completed self-report measures of cognitive ER and internalizing symptoms. Regression models controlled for internalizing symptoms, age, family income, and medication use. Sex did not moderate the association between ADHD status and maladaptive cognitive ER. In contrast, sex moderated the association between ADHD and adaptive cognitive ER ( *b* = -0.32, *p* = .045). Simple slopes analysis indicated ADHD status was associated with less frequent use of adaptive strategies among females ( *b* = -0.304, *p* = .011) but was unrelated for males ( *b* = 0.02, *p* = .882). Exploratory analyses of specific adaptive strategies indicated a significant ADHD × sex interaction for acceptance (i.e., acknowledging/coming to terms with what has occurred; *p* = .032), with the same pattern of simple slopes as observed for adaptive strategies. Findings suggest that ADHD-related differences in adaptive cognitive ER may be particularly relevant for females during early adolescence and highlight the importance of considering sex when examining ER in ADHD. Acceptance may warrant further investigation as a specific cognitive ER strategy relevant to females with ADHD.

